# Deep spatial attention networks for vision-based pavement distress perception in autonomous driving

**DOI:** 10.1371/journal.pone.0335745

**Published:** 2025-12-03

**Authors:** Fuwen Deng, Jiandong Jin

**Affiliations:** 1 School of Computer Science and Technology, Shandong Technology and Business University, Yantai, China; 2 School of Electronics Engineering and Computer Science, Peking University, Beijing, China; China Construction Fourth Engineering Division Corp. Ltd, CHINA

## Abstract

Ensuring the safety and comfort of autonomous driving relies heavily on accurately perceiving the quality of the road pavement surface. However, current research has primarily focused on perceiving traffic participants such as surrounding vehicles and pedestrians, with relatively limited investigation into road surface quality perception. This paper addresses this gap by proposing a high-performance semantic segmentation method that utilizes real-time road images captured by an onboard camera to monitor the category and position of road defects ahead of the ego vehicle. Our approach introduces a novel multi-scale spatial attention module to enhance the accuracy of detecting road surface damage within the traditional semantic segmentation framework. To evaluate the proposed approach, we curated and utilized a dataset comprising 2,400 annotated images for model training and validating. Experimental results demonstrate that our method achieves a superior balance between detection precision and computational efficiency, outperforming existing semantic segmentation models in terms of mean IoU while maintaining low computational cost and high inference speed. This approach holds great potential for application in vision-based autonomous driving as it can be seamlessly integrated with appropriate control strategies, thereby offering passengers a smooth and reliable driving experience.

## Introduction

The road network is a critical transportation infrastructure that serves as the pedestal for both manned and automated driving. However, the condition of pavements tends to degrade over time as a result of natural factors, such as erosion from freeze-thaw cycles, as well as dynamic loads imposed by traffic [1]. Pavement defects can significantly compromise both the safety and comfort of travel. For instance, a previous study demonstrated that a decrease in pavement smoothness resulted in a staggering 95.72% rise in accident rates on highways [2]. In Chicago, U.S., there were 11,706 reported complaints regarding pavement potholes within the first two months of 2018 alone [3]. In the realm of autonomous driving, despite the limited prevalence of large-scale applications at present, its penetration is steadily increasing. Recognizing that near real-time repairs are often impractical for most pavement failures, the implementation of road surface condition monitoring devices and utilizing the monitoring information for intelligent speed control and trajectory planning may enhance the safety and comfort of autonomous vehicles. Moreover, this benefit is amplified when considering connected autonomous vehicles (CAVs). Through collaborative driving automation, the pavement condition sensed by the front vehicles can assist the rear vehicles in making informed driving decisions in advance. This proactive behavior can effectively reduce congestion and traffic oscillations across the entire road network, further boosting the overall efficiency of CAVs [4]. Regrettably, the current research on autonomous driving environment sensing is primarily aimed at vehicle and pedestrian detection (e.g., [5–7]), with relatively little emphasis on road surface defect sensing. This dearth of research in the latter area continues to pose a significant challenge for real-time pavement condition sensing in the field of intelligent vehicles.

This paper aims to bridge this research gap by proposing a multi-categorization semantic segmentation model for recognizing pavement condition in autonomous driving. Our vision for the abovementioned pavement sensing encompasses the following principles:

1. Emphasizing the utilization of simple and cost-effective onboard sensors, such as the widely available video cameras, instead of relying on expensive and cumbersome high-precision specialized equipment (e.g., laser leveling meters). By prioritizing affordability and simplicity, we aim to enhance accessibility and adoption of pavement sensing technologies.
2. Enabling precise detection of pavement conditions at a fine-grained level, differentiating between various types of road surface defects including cracks, potholes, etc. This level of granularity facilitates distinct decision-making processes tailored to each specific condition, optimizing overall driving safety and comfort.
3. Advocating for algorithms with low computational complexity and advocating for a model architecture that is naïve and straightforward. This approach ensures seamless integration within the onboard computational devices while promoting energy efficiency, aligning with the goals of sus-tainable transportation.

To adhere to principle 1, we leverage the dashcam as the primary sensing device for data collection, eliminating the need for specialized pavement inspection equipment. In line with principle 2, our proposed method addresses pavement defect detection by framing it as a semantic segmentation problem in computer vision (CV). By performing pixel-level classification of pavement images and drawing on insights from pavement engineering studies (e.g., [8,9]), we effectively differentiate between various damage types. Thus, this approach offers abundant information about the road surface, aiding in decision-making for autonomous driving behaviors. For principle 3, we opted for a comparatively straightforward convolution-based neural network architecture instead of Transformer [10]. This alternative choice not only reduces the memory requirement but also alleviates the need for extensive training data.

Our contributions lie in proposing a multi-categorization semantic segmentation model for asphalt pavement distress detection, which leverages a convolutional attention mechanism. To ensure consistency and reliability of the results, the scope of this study is limited to daytime conditions with clear weather. To strengthen our findings, we conducted comprehensive field experiments to demonstrate the effectiveness of our approach. Regarding the model of neural network, our approach adopts the encoder-decoder architecture commonly used in semantic segmentation models [11,12]. To achieve high-accuracy recognition of diverse pavement defects, we harnessed the merits from existing research on semantic segmentation. This includes incorporating features such as multi-scale depth-separable convolution [13] attention mechanism [14], and all-MLP decoder [15], etc. Besides its application in pavement condition sensing for ego automated vehicles and CAVs, the proposed method holds promise for facilitating crowd-sourced pavement condition audit across the entire road network, enabling continuous health monitoring of road infrastructure.

The model’s output, i.e., a pixel-level segmentation mask, is directly usable by autonomous vehicle control systems. To enable actionable responses like speed adjustment and trajectory planning, an optional post-processing pipeline quantifies defect properties (e.g., area, width, severity) from the mask. These quantifications are passed to a decision-making module, realizing our vision of a proactive system that enhances safety and comfort.

The remainder of this paper is organized as follows:

- The *Related works* section provides a comprehensive overview of existing literature on pavement defect sensing.
- The *Methodology* section outlines our proposed model for pavement defect segmentation.
- The *Numerical experiments* section details the experimental setup and presents the corresponding results.
- The *Discussion* section offers an in-depth analysis of the findings in the context of prior research.
- The *Conclusion* section summarizes the key contributions of this study and outlines potential directions for future work.

## Goal and objectives

The primary goal of this study is to develop a cost-effective and efficient deep learning model for real-time, multi-category pavement distress segmentation, which can be deployed in vision-based autonomous driving systems. To achieve this goal, the following specific objectives have been established:

1. To curate a comprehensive dataset with multi-category, pixel-level annotations for various pavement distresses, including cracks, potholes, etc.
2. To design and implement a novel deep neural network architecture that strikes an optimal balance between high detection accuracy, low computational complexity, and high inference speed.
3. To validate the model’s performance using standard computer vision metrics such as mIoU, and to compare its performance against a comprehensive set of state-of-the-art semantic segmentation baselines.
4. To evaluate the model’s robustness under varying lighting conditions to demonstrate its adaptability to real-world scenarios.

## Background information

In recent years, an increasing number of studies have employed statistical machine learning and deep learning methods to identify pavement defects. Among these, computer vision-based approaches have shown particularly promising results [16]. Compared to conventional digital image processing techniques, they offer higher detection accuracy and improved adaptability to diverse environmental conditions [3]. Several open datasets have been made available to support research in this field, most notably the pavement damage detection dataset released by Hiroya et al. [17]. Since 2018, the authors have organized the Global Road Damage Detection Challenge (GRDDC) at the IEEE International Conference on Big Data to highlight the importance of machine learning–based approaches to road damage detection. The initial release of the dataset contained 9,053 images with 15,435 annotated pavement defects captured under diverse weather and illumination conditions, and it has since been expanded multiple times.

Broadly, techniques for processing pavement defect images can be categorized into three groups: (i) statistical machine learning methods or vanilla neural networks, (ii) detection-based methods, and (iii) segmentation-based methods. In this study, we provide a comprehensive review of these methodological developments as well as the associated datasets.

### Statistical machine learning methods and vanilla neural networks

Initial efforts to address pavement distress detection employed classical machine learning–based approaches. For instance, studies have utilized an image slicing method to enable approximate pavement condition assessment [18,19]. This technique involves dividing a full image into multiple small patches for classification, a task for which neural networks are leveraged to achieve high accuracy. In an earlier publication, Xu et al. [20] proposed an unsupervised pavement crack detection approach that leverages saliency map and statistical features. This method effectively identifies areas of crack by analyzing image intensity rarity and local contrast, while also evaluating the spatial continuity of potential cracks through neighborhood statistical features. Yousaf et al. [21] proposed a computer vision method using SIFT features with SVM classification and graph cut segmentation to detect and localize potholes in asphalt pavement images, achieving high accuracy. Hoang et al. [22] develops an intelligent approach that integrates image processing techniques with a multiclass support vector machine optimized by an artificial bee colony algorithm to automatically classify pavement cracks with high accuracy. In addition, classical statistical machine learning algorithms, e.g., Random Structured Forests [23], LightGBM [24], etc., have demonstrated their effectiveness in accurately identifying and classifying instances of pavement damage.

### Detection-based methods

Object detection is a fundamental task in the field of CV, focused on identifying and localizing multiple objects of inter-est within images or videos. Multiple classical algorithms have been developed for visual object detection, including well-established techniques, e.g., Faster R-CNN [25] and YOLO [26]. Du et al. [27] proposed a YOLO-based approach to enable automated pavement distress detection. The study showcases commendable accuracy and inference speed achieved by the proposed method, concurrently examining the optimal illumination upon which the algorithm depends. Zeng et al. [28] developed a lightweight road damage detection model based on YOLOv8n. In this study, the large separable kernel attention mechanism is introduced to enhance the detection accuracy. Similar studies [29,30] have been conducted using an updated iteration of the YOLO approach. Ju et al. [31] proposed CrackDN, a model developed on the foundation of the Fast R-CNN framework, with the purpose of effectively detecting both sealed and unsealed pavement cracks. This approach combines a sensitivity detection network and a feature extraction convolutional neural network in parallel. Notably, the method accommodates challenging background scenarios, such as unbalanced illumination and road markings, setting it apart from alternative object detection models like Faster R-CNN and SSD300 while consistently achieving superior performance. Yang et al. [32] made enhancements to the feature extraction network in the context of pavement crack detection. Their improvements involved introducing a feature pyramid and hierarchical boosting network to enrich the low-level features with contextual information, and a nested sample reweighting method to boost the performance specifically for hard samples. Likewise, Naddaf-Sh et al. [33] introduced the bi-directional feature pyramid network as a means to enhance the accuracy of pavement crack detection.

To improve the adaptability of the model, recent studies have made significant advancements in refining model architectures. For example, Zhang et al. [34] introduced a novel road damage detection algorithm, termed FPDDN, which incorporates a deformable transformer, a D2f block, and an SFB module to effectively predict pavement damage of varying sizes through a multi-branch framework. Similarly, Lin et al. [35] employed unsupervised domain adaptation techniques to enhance the generalization capability of road damage detection models, enabling robust performance across diverse geographical regions. These innovations demonstrate the potential for achieving greater adaptability and accuracy in model applications.

### Segmentation-based methods

In contrast to object detection, which typically labels images using bounding boxes around specific objects, semantic segmentation provides a more detailed output by assigning a class label to each pixel in the image [12]. Studies in literature have utilized semantic segmentation techniques to achieve pixel-level segmentation of road surface images. For instance, Sudhir et al. [36] developed a city-scale road audit system, wherein they introduced a semantic segmentation network based on the ERFNet architecture. This network was specifi-cally designed to accurately localize road surface defects, enabling precise identification and characterization of such anomalies. Along similar lines, Liu et al. [37] presented a se-mantic segmentation model for end-to-end pavement crack detection, employing an encoder-decoder model architecture. Zhang et al. [38] introduced CrackNet, a convolutional neural network-based model designed for pavement crack detection. This model utilizes an FCN-like structure [39] to achieve pixel-level extraction of cracks, resulting in a high-precision and high-recall detection performance. The researchers made further improvements to the model, which encompassed adopting a segmentation network architecture utilizing recurrent neural networks (RNN) [40] and designing a specialized crack detection model specifically tailored for 3D images. There also exist studies (e.g., [41–43]) wherein researchers have employed U-Net based network architecture to achieve pixel-level segmentation of pavement cracks, demonstrating highly promising outcomes. Majidifard et al. [44] curated a comprehensive pavement distress dataset utilizing Google Street View images. They employed both YOLO and U-Net models to process the dataset, integrating the results to effectively categorize and quantify the severity of each observed distress condition. In a related effort, Ye et al. [45] presented a instance segmentation model to provide accurate pavement crack identification results based on YOLOv7 network. Mei et al. [46] introduced the ConnCrack model, which integrates a conditional Wasserstein GAN model with a connectivity map for robust road crack detection. Zhang et al. [47] suggested a novel supervised generative adversarial learning technique that effectively mitigates the “All-black” phenomenon in pavement crack segmentation task. Such phenomenon pertains to erroneously labeling all pixels as background due to unbalanced samples. Liu et al. [48] introduced a pavement crack segmentation algorithm named CrackFormer, which integrates Transformer encoder modules into the existing segmentation framework to enhance its performance.

### Available datasets

We also reviewed existing pavement distress datasets, summarized in Table 1. Consistent with the focus of this paper, our review primarily covers computer vision datasets, which can be broadly categorized into three types: image classification, object detection (with bounding boxes), and pixel-level mask annotation.

**Table 1:** Available pavement condition datasets.

Dataset	Annotation Type	Descriptions
Majidifard Dataset [49]	Bounding box	Containing 7,237 pavement images collected based on Google Street View images.
GAPs v2 [18]	Classification tag	Containing 2,468 grayscale road surface images annotated with Boolean value for whether it is a pavement distress area.
AEL [50]	Single-category mask	A total of 269 real road surface images annotated with masks of crack areas.
CRACK500 [51]	Single-category mask	Encompassing 500 labelled pavement images labelled with crack masks.
CFD [23]	Single-category mask	Including 118 pavement images collected in China and manually annotated with crack masks.
CrackTree Dataset [52]	Single-category mask	Including 206 pavement image data labelled with crack masks.
GRDDC2020 [17]	Bounding box	Including over 20,000 images from Japan, the Czech Republic and India.
CrackDataset [31]	Single-category mask	Containing 3,000 road surface images labelled with crack masks.
RTK [53]	Classification tag	Containing 6,264 images of road surfaces labelled with gradings of pavement conditions.

The existing body of literature has established a robust foundation for automated pavement distress detection. However, there remains a critical gap in a single, comprehensive solution that combines the benefits of fine-grained, multi-category pixel-level segmentation with the real-time, low-cost demands of on-board autonomous vehicle applications. Most semantic segmentation datasets are limited to single distress categories, primarily cracks, and often utilize high-cost imaging systems. The challenge lies in creating a unified framework that can accurately distinguish a variety of distress types using simple hardware, while also being computationally efficient enough for real-time processing in a consumer vehicle. This paper aims to fill this void by proposing such a multi-categorization semantic segmentation model.

## Methodology

This section introduces the proposed multi-categorization semantic segmentation model for road surface defect sensing in the context of vision-based autonomous driving systems. We commence by discussing the categorization of road surface defects, aligning with the consensus within the field of pavement engineering, and subsequently introduce the specific objects targeted for identification in this study. Our focus then transitions to the comprehensive architecture of the employed model, offering detailed insights into the design of the encoder, convolutional spatial attention mechanism, and finally, the decoder.

**Fig 1:**
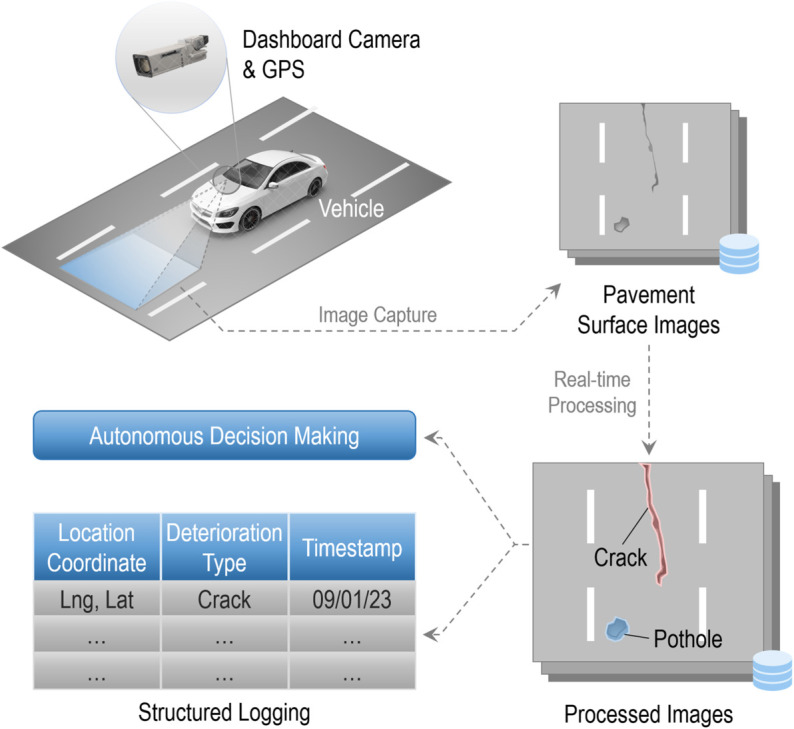
Schematic of the workflow. The figure demonstrates a typical workflow of onboard road surface monitoring in vision-based autonomous vehicles.

Fig 1 illustrated the workflow of a generalized self-driving pavement sensing system. Initially, a forward-facing dashcam mounted on the vehicle continuously captures images at a pre-determined distance and stores them in cache. Subsequently, the captured images are processed using a dedicated model, resulting in outcomes with pavement defect masks. This output serves two purposes. Firstly, it informs behavioral decision-making of the autonomous driving, contributing to tasks such as speed control and trajectory planning. Secondly, the outcomes are stored in a spatial database, forming structured logs to facilitate ongoing monitoring of road quality in CAV environment. The primary objective of this paper is to develop the real-time image processing model as above that fulfills the demands of pavement distress segmentation.

### Categorization of road surface defects

A comprehensive examination of roadway distress plays a crucial role in data engineering and modeling endeavors. It is evident that not all pavement defects can be treated equally due to their distinct geometries, structural properties, and impacts on vehicle travel. Consequently, one of the foremost challenges in visual pavement monitoring lies in effectively categorizing these distresses. In pavement engineering, the prevailing types of distress commonly encountered on asphalt pavements encompass the following [8]:

(1) **Longitudinal and transverse cracking.** Longitudinal cracking transpires when cracks develop parallel to the direc-tion of traffic flow, while transverse cracking emerges when cracks are oriented perpendicularly to the pavement’s center-line. The causes encompass a variety of factors, including pavement cracking resulting from temperature fluctuations, fatigue cracking occurring in the wheel track belt due to repet-itive traffic loads, uneven settlement of the roadbed, and the presence of reflection cracks triggered by old pavement underneath.

(2) **Alligator cracking.** Alligator cracking, characterized by an interlaced pattern that resembles the scales of an alligator’s back, is also a common distress in pavement engineering. This phenomenon can be attributed to clear factors, specifically structural fatigue damage caused by the repeated traffic loads.

(3) **Ravelling.** Ravelling is a term that refers to the gradual disintegration and loss of aggregate particles from the surface of an asphalt pavement. The formation of ravelling can be attributed to inadequate asphalt adhesion, degradation of the asphalt material, or the repeated occurrence of freeze-thaw cycles on the pavement surface.

(4) **Pothole.** Potholes are the bowl-shaped depressions in the pavement surface. They arise from a localized loss of aggregate within the pavement. It typically originates from various conditions, including alligator cracking, ravelling, and other related factors.

(5) **Rutting.** Rutting refers to the pavement surface depres-sion and uplift along the wheelpath. This type of distress can be attributed to factors such as the cumulative deformation of the roadbed under long-term traffic loads and the flow and raveling of asphalt concrete caused by high temperatures.

This study focuses on the identification of three major pavement distress types: (i) longitudinal and transverse cracks (collectively referred to as “cracks”), (ii) alligator cracks, and (iii) potholes. These distresses were selected due to their prevalence across diverse regions. In addition to primary distresses, it is also essential to recognize repaired areas. Although repair treatments are applied, such areas remain potential weak points in the pavement structure. Moreover, repairs often create vertical displacements relative to the normal pavement surface, adversely affecting driving comfort. Accordingly, this study highlights two representative repair types, i.e., crack sealing and asphalt patching, as case studies for identification and analysis. Equally important is the detection of structural elements that deviate from standard pavement surfaces, such as utility covers and bridge expansion joints. These features can also produce vertical displacements that compromise ride quality, as exemplified by the common phenomenon of “bridge jumping” when traversing expansion joints. While previous studies (e.g., [54]) have emphasized the detection of road markings, we contend that markings are integral components of the road surface and do not substantially affect safety or comfort. Hence, they are excluded from additional labeling in this research.

**Fig 2:**
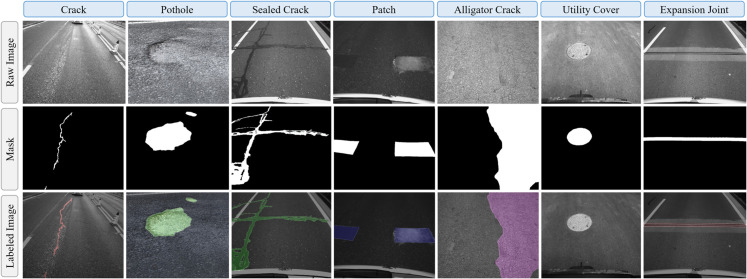
Primary items involved in vision-based pavement condition monitoring. The figure illustrates the seven types of pavement damage considered in this study, including the original images and their corresponding annotated segmentation masks.

As illustrated in Fig 2, seven categories of pavement-related objects are considered in this study. Using semantic segmentation models, we provide pixel-level mask annotations for each category to ensure accurate labeling.

### Datasets

The dataset utilized in this study was captured using a vehicle-mounted camera and annotated with multi-category masks. This approach was chosen because, from both a data collection and annotation perspective, it strikes an effective balance between detection accuracy, speed, and cost, in the context of fine-grained pavement inspection.

Regarding data collection methods, existing approaches include drones, depth cameras, LiDAR, smartphone vibration sensors, etc. Depth cameras and laser leveling instruments offer high detection accuracy (on the millimeter scale) and can provide detailed pavement inspection results. However, these methods are costly and have slow detection speeds, which limits their scalability. For example, laser leveling equipment operates at a slow pace and is typically used only for post-construction quality assessments. Drones and smartphones offer broader coverage but lack the precision needed for detailed analysis. Drone images often do not meet the required resolution, while smartphone data can only estimate road flatness at the section level, making it challenging to pinpoint the exact location and type of pavement damage. In contrast, the vehicle-mounted camera-based pavement inspection method offers a more favorable balance of data collection cost, detection speed, and accuracy. With the increasing penetration of CAVs, this approach holds potential for automated data collection and better compatibility with V2X systems, enabling large-scale applications.

To meet the demand for fine-grained identification of pavement defects in real-world pavement management, we employ multi-category mask annotation for data labeling. Compared with traditional bounding box annotation, this approach enables a more comprehensive evaluation of pavement distresses by simultaneously capturing factors that are critical for informed maintenance decision-making, e.g., the type of damage, the affected area, and its severity. Such detailed annotations facilitate processes such as selecting appropriate maintenance strategies, accurately estimating material requirements, and improving the overall performance of pavement management systems. To the best of our knowledge, the dataset developed in this study is among the first pavement condition datasets to incorporate multi-category labels. The current version comprises 2,400 images, specifically curated to support the development of advanced, high-accuracy models.

**Fig 3:**
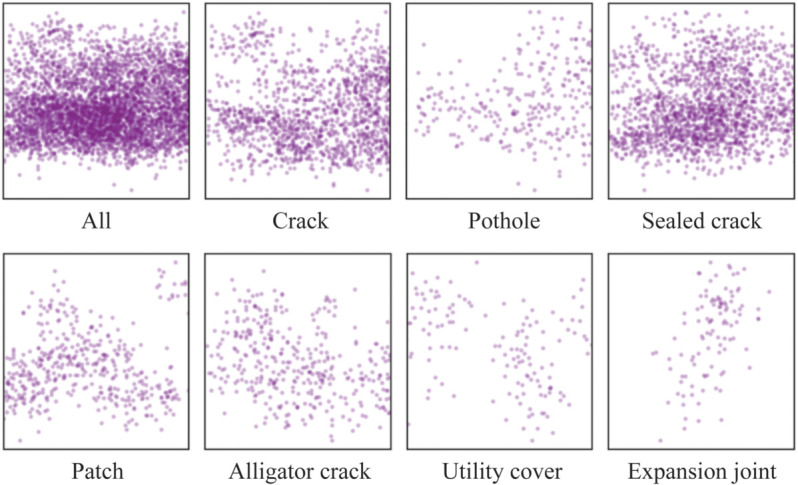
Segmentation mask center distributions of the dataset. The figure presents the geometric centers of the segmentation masks for all annotated pavement distresses in the dataset, as well as the centers corresponding to each specific category.

In constructing the dataset, we did not perform cropping to position the masks at the center of the images; rather, we preserved the original distribution of the masks within the image plane to align with the real-world context of pavement health monitoring. The distribution of centroids for all segmentation masks within the dataset is illustrated in Fig 3. It reveals that the distribution of mask centroids is notably discrete. The mean and standard deviation of the normalized distance between the center points of the masks and the center of the image plane are 0.379 and 0.153, respectively.

### Image de-noising

In the raw images collected, various types of noise may be present. Examples include overexposure or underexposure caused by variations in ambient light intensity, motion blur resulting from excessive vehicle speed, and shadows cast by roadside trees or buildings that interfere with the recognition of pavement distresses. Different strategies were adopted to address these sources of noise.

**1. Variation in ambient illumination** Differences in ambient light intensity result in noticeable brightness variations across the collected images. Generally, images captured under moderate lighting conditions exhibit balanced brightness, appropriate contrast, and a relatively high dynamic range, making them suitable for pavement distress detection. In contrast, images that are excessively bright or dark reduce detection accuracy. To mitigate this issue, we leverage the generalization capability of the model. Specifically, by incorporating training data with diverse illumination conditions, the model can better adapt to brightness variations, thereby reducing the negative impact of ambient light on recognition accuracy.

**2. Motion blur** Even with the camera parameters configured, motion blur may still occur due to the high velocity of the vehicle. To mitigate this effect, we employed conventional digital image processing techniques. Specifically, blurred images were identified using the Laplacian variance method, which quantifies the focus of an image by computing the variance of the Laplacian response across its pixels. The procedure is described as follows:

$$Lap(x,y)=\nabla^{2}I(x,y),\;Var=\frac{1}{N}\sum_{i=1}^{N}(L_{i}-\mu {)}^{2},$$ (1)

where *Lap*(*x*, *y*) denotes the Laplacian response of the image pixels, *μ* is the mean, and *Var* represents the variance of the Laplacian response.

Subsequently, Wiener filtering can be applied for deblurring:

$$\hat{I}(x,y)={ℱ}^{-1}\left[\frac{O^{*}(u,v)*O(u,v)}{|O(u,v){|}^{2}+K}\right]$$ (2)

where $\hat{I}$ is the deblurred image; ${ℱ}^{-1}$ denotes inverse Fourier transform; *O* and O^*^ denote the optical transfer function and its complex conjugate, respectively; *G* represents the frequency-domain representation of the original image; *K* corresponds to the signal-to-noise ratio (SNR).

**3. Shadows in images** For shadow removal, we adopted a GAN-based method from our previous work [55]. This method can be incorporated as a standard pre-processing module within the preprocessing pipeline.

### Semantic segmentation model

This paper tackles the issue of road surface perception in vision-based autonomous driving systems through the utilization of semantic segmentation approach. The application of semantic segmentation is justified for two main reasons. Firstly, pavement defect expands freely on the road surface without a clear boundary, making the concept of “boundary" redundant and meaningless for detecting pavement defect using object detection or instance segmentation techniques. Secondly, in comparison with object detection models, employing semantic segmentation methods allows for a more accurate assessment of the area covered by different types of pavement defects, providing more comprehensive and informative results.

The semantic segmentation task for pavement distress identification can be formally stated as follows. Given a sample set $S={\left\{\left(x_{s},y_{s}\right)\right\}}_{s=1}^{N_{s}}$ with *N*_*s*_ labeled images. Herein, *x*_*s*_ represents the sample pavement image with dimensions [*H*,*W*,*C*] (height, width, and number of channels), and in the case of a general color image, *C* = 3. The labeled mask information associated with *x*_*s*_ is denoted as *y*_*s*_, with dimensions [H,W,T  +  1], where *T* represents the number of categories of interest for pavement distresses, with the inclusion of an additional 1 to account for the background of images. Based on the aforementioned categorization approach for pavement distresses, there are a total of *T* = 7 distinct categories. Our objective is to construct a semantic segmentation model $F_{\Theta }$, utilizing *S*, that can accurately predict masks for *T* categories of pavement distresses. That is, we aim to learn a mapping as follow:

$$S\to F_{\Theta }(•|S),$$ (3)

then, by feeding an arbitrary pavement image *x*_*q*_ with the same dimensions as the sample image into the model $F_{\Theta }$, we can obtain the predicted mask region ${\hat{y}}_{q}$ for various pavement distress categories, i.e.:

$${\hat{y}}_{q}=F_{\Theta }(x_{q}|S).$$ (4)

**Fig 4:**
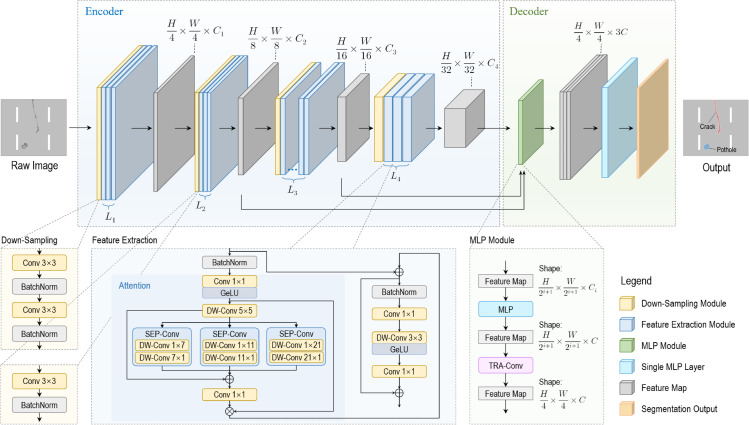
Neural network architecture of the utilized semantic segmentation model.

Consistent with most prior research (e.g., [11–13]), we adopted the widely employed encoder-decoder architecture, which has proven effective in semantic segmentation tasks. The proposed model architecture is demonstrated in Fig 4. In detail, the deep neural network is composed by an encoder *E* and a decoder *D*, so the semantic segmentation model can be represented as:

$$F_{\Theta }(•|S)=D(E(•);S).$$ (5)

In this model, a powerful image encoder is employed to extract multi-scale features from the raw image. Subsequently, the decoder utilizes these features to generate accurate masks highlighting areas of pavement distress regions. Depth-wise convolution [56] and spatially separable convolution [57], denoted as DW Conv and SEP Conv respectively in Fig 4, are employed to reduce both the number of parameters and the computational cost of floating-point operations in the network.

This study utilizes input and output images with a height and width of predefined fixed numbers of pixels, i.e., *H* and *W*. If the dimensions of the input pavement surface image differ from these specifications, resizing the image to match the aforementioned dimensions is necessary. In line with the majority of classification models, we adopt the cross-entropy loss function in our approach. Specifically, for the task of pavement distress segmentation, we denote the loss function as:

$$Loss(x^{q},y^{q})=-\frac{1}{H\times W}\sum_{p}\sum_{u}y_{p,u}^{q}\ln F_{\Theta }(x_{q}|S{)}_{p,u},$$ (6)

where *p* denotes a certain coordinate point, or a pixel, on one image, *u* represents the index of considered categories of pavement distresses.

We have incorporated many of the successes of prior stud-ies to achieve a balance of accuracy and efficiency in the model. To enhance accuracy, we implemented multi-scale feature fusion along with spatial attention mechanisms. On the other hand, to improve efficiency, we employed a set of simple convolution-based modules, separable convolution, and a compactly structured decoder.

### Encoder and convolutional attention module

To achieve multi-scale feature extraction, we adopt a similar encoder architecture formed in previous studies [13,15] by utilizing multi-stage feature extraction. The feature extraction process comprises four stages, with each stage resulting in a feature map with the size of $\frac{H}{2^{i+1}}\times \frac{W}{2^{i+1}}\times C_{i}$ after the *i*-th stage. Note that *H* and *W* represent the height and width of the input image, respectively. The number of channels in each stage of the output feature map is a manually set parameter. The encoder follows a modular architecture, where each stage consists of a down-sampling module and *L*_*i*_ convolutional attention modules in the *i*-th stage. The down-sampling module comprises a single 3×3 convolutional followed by a batch normalization operation, which both need to be repeated one additional time during the first stage. We used GeLU [58] activation function in our approach.

In the context of pavement damage identification, the morphology of pavement damage significantly differs from that of general objects. A distinctive characteristic is that pavement damage can manifest in various forms, such as block-like shapes (e.g., patches or potholes with moderate aspect ratios) or elongated shapes (e.g., cracks). Additionally, pavement damage exhibits strong scale heterogeneity, ranging from localized regions to areas spanning the entire image. To address these characteristics, the model must possess two key capabilities: (1) the encoder should be capable of effectively learning representations of stripe shapes with irregular aspect ratios; (2) a multi-scale feature extraction strategy is required to accurately identify pavement damage across varying scales.

**Fig 5:**
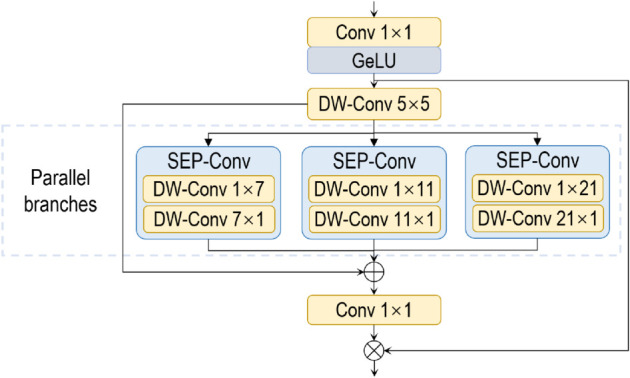
Diagram of convolutional attention module.

To achieve these objectives, we have strategically incorporated a convolutional attention module, which serves as a spatial attention mechanism in a compact form. The module is composed of a series of parallel variable-size separable convolutional modules, as demonstrated in Fig 5. These modules consist of deepwise stripe convolution operations, with kernel sizes of 1×m and m×1. The mathematical formulation of the attention mechanism is as follows:

$$\text{Att}={\text{Conv}}_{1\times 1}\left(\sum_{i=0}^{3}{\text{Scale}}_{i}(\text{DWConv}(F))\right),$$ (7)

where *F* is the input feature, Att is the attention map, and ${\text{Scale}}_{i}$ for i∈{0,1,2,3} denotes the *i*th branch. The ${\text{Scale}}_{0}$ branch is an identity connection that bypasses the convolutional operations, thereby preserving the original feature information. This architecture effectively provides multi-scale context from local to global, ensuring adaptability in both spatial and channel dimensions.

The design is specifically intended to capture elongated structural features, such as strip-shaped fractures that commonly characterize pavement cracks. Moreover, the use of parallel modules enables simultaneous multi-scale feature extraction, which is essential for accurately detecting pavement distresses that may range from small, localized defects to extensive areas covering the entire image.

### Decoder

Research has shown that encoders require a strong feature extraction capability, which typically necessitates deeper neural networks. In contrast, decoders can employ lightweight structures to alleviate the computational load. Therefore, we employ the All-MLP decoder proposed in [15]. The decoder first uses multilayer perceptron (MLP) to unify the channel sizes of the extracted feature maps to *C*. It then unifies the feature map height *H* and width *W* through transposed convolution operations. Next, it concatenates the feature maps and generates segmentation results via another MLP. Finally, a bilinear up-sampling operation can be con-ducted to restore the resolution to that of the input images. The All-MLP decoder is a deliberate design choice that complements the powerful encoder by focusing on feature aggregation rather than a complex fusion of information from multiple, distinct scales as seen in architectures like U-Net. This architectural choice represents a balance between the need for an accurate representation of fine details and the critical requirement for real-time computational efficiency.

## Results and discussion

To evaluate the efficacy of the proposed approach, we conducted numerical experiments and compared its performance with that of other existing models. Moreover, we assessed the model’s capability to identify various types of pavement distresses based on experimental results.

### Implementation details

Given the limited availability of multi-category pavement distress segmentation datasets, we constructed a dataset of 2,400 pavement images for training and validation in this study. The dataset is publicly accessible at https://github.com/FuturePave/PaveSeg-Dataset. Each image contains at least one instance of pavement distress. Of these, 1,500 images were collected and annotated by our team, while 700 images were sourced from the GRDDC dataset [17] and 200 from the CrackTree dataset [52].

The inclusion of external data serves two purposes: (i) enhancing environmental diversity and (ii) balancing the distribution of distress categories. Specifically, the GRDDC dataset contributes a multinational scope, encompassing diverse pavement types and conditions, while the CrackTree dataset addresses class imbalance by providing a large number of high-quality crack images. All external data were carefully filtered to meet resolution requirements, and additional pixel-level distress masks were created to ensure accurate labeling.

To acquire the self-collected data, we utilized a dashcam mounted on the front of a car. During the recording process, we adjusted the dashcam settings to a high frame rate and low exposure time mode. In detail, the dashcam was set to a frame rate of 30 FPS, with an exposure time of 20 ms. While a relatively low frame rate would suffice for ensuring longitudinal spatial coverage of the roadway, a higher frame rate is selected to account for variations in the probe vehicle’s speed and to minimize the detrimental effect of potential motion blur due to prolonged exposure time. The detailed camera parameters and configuration employed in the experimental setup are provided in Table 2. The data collection process involved capturing images under varying lighting conditions. Furthermore, we conducted a diligent screening of the collected data, taking into account the diverse range of pavement distress types.

**Table 2:** Camera parameters and configuration.

Component	Specification	Details
Camera	acA2500-60uc	Front-facing, high-resolution color camera.
Maximum resolution	2592 × 2048px	Capable of capturing high-resolution imagery.
Lens focal length	16mm	Fixed lens, wide-angle.
Mounting	Windshield mount	Positioned behind the rearview mirror.
Tilt angle	40^°^	Calibration is required based on the specific vehicle.
Data capture	30 FPS, 20 ms	High frame rate to minimize motion blur.

We allocated 1,700 images from this dataset to the training set, 300 images to the validation set, and reserved the remaining 400 images as the testing set. In our experiments, we selected an input image size of 512×512 pixels, resulting in model input dimensions of 512 for both height (*H*) and width (*W*). This choice achieves a balance between computational efficiency and recognition accuracy.

To enhance the generalization capability of our model, we employed image preprocessing techniques and implemented image augmentation strategies. Firstly, we cropped the images in the dataset to exclude the off-road region, specifically focusing on removing the hood of the vehicle. Subsequently, we conducted image data augmentation using the Augmentor tool. Various augmentation approaches including rotating, random cropping and perspective skewing were applied. Furthermore, we expedited the model training process by leveraging pre-training on a dedicated surface defect segmentation dataset [59]. This step effectively reduced the overall training time required for the model training task.

For the encoder, at the *i*-th stage, the number of channels is set to *C*_1_ = 64, *C*_2_ = 128, *C*_3_ = 320, and *C*_4_ = 512. The number of convolutional attention modules is defined as *L*_1_ = 3, *L*_2_ = 3, *L*_3_ = 12, and *L*_4_ = 3, respectively. For the depthwise stripe convolution operations, the hyperparameter *m* is assigned values of 7, 11, and 21. In the decoder, the channel dimension of the extracted feature maps is fixed at *C* = 256.

Typically, in image detection or segmentation tasks, a batch size of 1 is applied during the inference stage to support frame-by-frame processing. However, this approach may result in data I/O becoming a significant bottleneck, limiting inference efficiency. To address this, we leveraged the parallel processing power of the GPU. Specifically, we developed an image buffer to optimize processing. Rather than performing inference on a frame-by-frame basis, we accumulated a set of frames from the video stream captured by the camera into the buffer, and then processed them as a batch on the GPU. Experiments were conducted to evaluate the impact of this batch processing strategy on inference performance.

### Performance comparison

**Fig 6:**
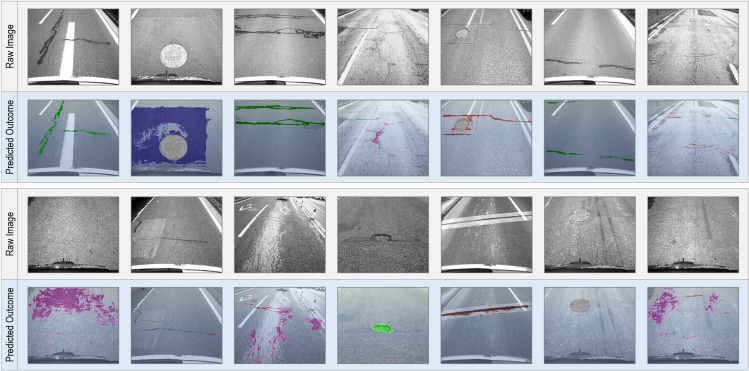
Examples of pavement distress segmentation model outcomes.

Fig 6 demonstrates the effectiveness of the trained pavement segmentation model when applied to a subset of the validation set images. The results in Fig 5 highlight the model’s capability to accurately distinguish between various types of pavement distresses. Notably, the model exhibits proficiency in identifying complex crack morphologies and scenarios where multiple objects are simultaneously present. And it is worth highlighting that the identification of alligator cracks can effectively distinguish them from other cracks, despite their similar local morphology. This distinction emphasizes the significant contribution of multiscale features in accurately discerning between these two types of distresses.

Consistent with the prevalent research in semantic segmentation, we assess the efficacy of our pavement distress segmentation model using the mean Intersection over Union (*mIoU*) metric. The *mIoU* metric, defined as:

$$mIoU=\frac{1}{T+1}\sum_{u=1}^{T+1}\frac{n_{uu}}{\sum_{v=1}^{T+1}n_{uv}+\sum_{v=1}^{T+1}n_{vu}-n_{uu}},$$ (8)

serves as a reliable measure to gauge the accuracy and quality of our model’s segmentation predictions. In Eq 8, *u* and *v* denote the indices of considered categories of pavement distresses, and $n_{uv}$ represents the count of pixels that are classified as category *v* while belonging to category *u*. We may also evaluate the *IoU* metric for one category *u* individually, defined as:

$$IoU_{u}=\frac{n_{uu}}{\sum_{v=1}^{T+1}n_{uv}+\sum_{v=1}^{T+1}n_{vu}-n_{uu}}.$$ (9)

This allows us to assess the model’s performance in recognizing specific types of pavement distresses.

We employed several classical models, namely FCN [39], Unet++ [60], Deeplabv3+ [61], PSPNet [62], EMANet [63], MaskFormer [64], SETR [65], Mask2Former [66] and PIDNet [67] as baselines. We also compared lightweight models, including MobileNetv2 [68] and EfficientNet [69] with DeepLabv3+ segmentation head, to evaluate the performance of the proposed model against such compact architectures.

All baseline models underwent the same pre-processing and pre-training procedures as our model. The inference experiments were consistently conducted on a unified computing platform equipped with an Intel Core i9-14900K CPU and an NVIDIA RTX 4090 (24GB) GPU. The performance evaluation results for both our model and the baseline models, using the mIoU metric, computational complexity and inference speed as indicators of model accuracy and efficiency, are presented in Table 3. In this table, we provide details including model name (with the utilized backbone network), the number of parameters (in millions), the number of floating-point operations (in GFLOPs), the corresponding mIoU metric values, and frame-by-frame inference speed (in FPS).

**Table 3:** Performance comparison with baselines.

Model (Backbone)	#Params (M)	FLOPs (GFLOPs)	mIoU (%)	#FPS
FCN (ResNet-101)	45.7	42.8	65.0 (–3.7)	57.3
Unet++ (Resnet-101)	59.5	56.4	65.3 (–3.4)	49.2
Deeplabv3+ (Resnet-101)	58.7	55.1	68.1 (–0.6)	51.7
PSPNet (ResNet-101)	48.2	51.5	67.9 (–0.8)	56.0
EMANet (ResNet-101)	53.6	52.0	68.3 (–0.4)	53.8
MaskFormer (ResNet-101)	65.8	61.9	68.5 (–0.2)	43.1
SETR-MLA (T-Base)	47.0	44.1	68.1 (–0.6)	56.9
Mask2Former (ResNet-101)	64.7	59.2	68.5 (–0.2)	45.6
PIDNet (PIDNet-L)	27.3	45.0	68.2 (–0.5)	52.3
Deeplabv3+ (MobileNetv2)	6.9	4.8	48.1 (–20.6)	117.8
Deeplabv3+ (EfficientNet)	7.4	5.3	51.4 (–17.3)	108.5
Ours	27.8	41.6	68.7	58.9

The results demonstrate that our model outperforms baseline models in terms of achieving the highest mIoU metrics (68.7%) while achieving a relative high processing speed (58.9 FPS). Lightweight models, such as those based on MobileNetV2 or EfficientNet architectures, achieved superior inference speeds but exhibited significantly lower mIoU scores than their larger counterparts. This performance trade-off stems from their constrained model capacity, a direct consequence of lightweight design, which impedes the preservation of fine-grained spatial details.

To assess practical deployment potential, inference speed was evaluated on an embedded platform (NVIDIA Jetson Xavier NX, 8GB). A throughput of 16.2 FPS was achieved, indicating that the model not only meets real-time processing requirements but is also a promising candidate for integration into lightweight vehicle-mounted systems.

### Per-class performance breakdown

To provide a more granular evaluation, a comparative analysis of model effectiveness in identifying various pavement distress types was conducted. In Table 4, we present the *IoU*, precision and recall metrics for each category alone (excluding the background class), as well as *IoU* deviation values compared to *mIoU*. The results obtained indicate that the *IoU* of pavement surface objects with clear boundaries, e.g., utility covers and expansion joints, are higher than *mIoU*, reaching 87.3% and 71.6%, respectively. In contrast, the segmentation outcomes for sealed cracks are better than that of cracks and alligator cracks because the boundary with normal pavement is more distinguishable. For crack and alligator crack, the recall is notably higher than the precision. This indicates that the model is effective at identifying a high percentage of these defects (i.e., it doesn’t miss many of them), but it also produces a significant number of false positives (i.e., it sometimes labels normal pavement as a crack). Conversely, for objects like Utility Covers and Expansion Joints, both precision and recall are high, indicating a reliable and accurate performance.

**Table 4:** Comparison of different categories.

Pavement Distress Type	IoU (%)	Precision (%)	Recall (%)	Deviation (%)
Utility Cover	87.3	91.2	90.5	18.6
Expansion Joint	71.6	75.8	74.3	2.9
Pothole	64.5	68.1	67.9	–4.2
Sealed Crack	62.4	67.5	65.2	–6.3
Patch	59.7	64.9	63.1	–9
Alligator Crack	58.4	62.3	66.8	–10.3
Crack	57.7	60.1	63.4	–11
(Mean)	68.7	70.2	70.0	0

### Robustness under various lighting conditions

Accurate detection of pavement damage is challenging due to variations in illumination, highlighting the need for a comprehensive evaluation of the model’s performance under different lighting conditions. However, directly measuring ambient illuminance during data collection is often impractical. To assess and compare the model’s performance across varying lighting scenarios, we use perceived brightness, a widely accepted metric in image processing, as an indirect measure of lighting conditions. The calculation of perceived brightness is given by the following equation:

$$P=\frac{0.299R+0.587G+0.114B}{2.55},$$ (10)

where *R*, *G*, and *B* represent the normalized red, green, and blue color values of the pixel, respectively, when the RGB color model is employed. This approach involves calculating the average perceived brightness of all pixels within a given image, providing a useful indication of the lighting environment during data collection. Alternatively, for data captured by black-and-white cameras, the average pixel gray value of the image may be used directly as a substitute.

**Fig 7:**
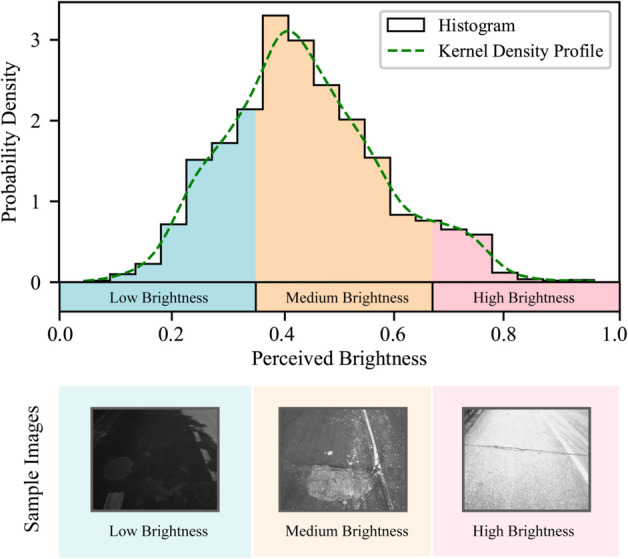
Luminance classes of sample images.

Using this approach, we calculated the average perceived brightness of all sample images. The histogram of the probability distribution of the average perceived luminance for these images is shown in Fig 7. As illustrated, we selected the 33.3% and 66.7% quantiles of perceived brightness (0.350 and 0.657) as threshold points to categorize the sample images into three luminance classes: low, medium, and high. Sample images from each luminance category are also provided in Fig 7. Generally, low brightness is associated with cloudy weather conditions or shadow occlusion, while high brightness typically results from camera overexposure. Since the training, validation, and test sets were randomly sampled from the full set of images, their luminance distributions are similar to that of the entire dataset.

During training, the full training set was used without differentiating between brightness levels. In the testing phase, we compared the model’s performance on images from each luminance class. The results, presented in Table 5, show that while medium luminance achieves the highest accuracy, the performance on low- and high-luminance images does not exhibit significant degradation. Specifically, the accuracy for low-luminance images is only 0.9% lower, and for high-luminance images, it is 0.4% lower than the overall average. These results suggest that the model demonstrates robustness to variations in lighting conditions. Furthermore, to address the degradation observed for low-brightness images, a promising avenue for future improvement could involve incorporating low lighting image enhancement techniques in the preprocessing stage, which have been shown to enhance illumination adaptability in previous studies [70,71].

**Table 5:** Performance evaluation across luminance classes.

Luminance classes	Quantiles	mIoU (%)
Low Brightness	0-33.3%	67.80%(–0.9%)
Medium Brightness	33.3%-66.7%	69.10%(+0.4%)
High Brightness	66.7%-100%	68.30%(–0.4%)
All	0-100%	68.70%

### Ablation study

To evaluate the effectiveness of the applied convolutional attention module, i.e., parallel branches of separable stripe convolutions, we perform a controlled ablation study that isolates the contribution of this module to segmentation accuracy and runtime performance. In detail, we define and implement the following model variants to isolate module effects:

1. Baseline: backbone network with the decoder (no stripe branches);
2. Stripe-DW: add parallel depthwise stripe branches for m∈{7,14,21}, fuse by concatenation followed by 1×1 pointwise convolution;
3. Stripe-DW (no fusion): same as Stripe-DW but without the final fusion;
4. Single-scale stripe: *m* = 21 branch only.

Table 6 summarizes the ablation results. Adding the parallel stripe branches yielded a significant improvement in mIoU (+1.4%) compared with the baseline, while incurring a small additional inference cost (+1.2 FPS). Replacing the stripe branches with a single large 21 × 21 depthwise convolution achieved a smaller gain (+0.9%) with close inference speed, indicating that the separable stripe design offers a superior accuracy-to-computation trade-off. Ablations removing the final 1×1 fusion showed smaller improvements, demonstrating that channel fusion is beneficial for integrating multi-scale stripe responses.

**Table 6:** Ablation results comparing parallel separable stripe convolution variants.

Variant	*mIoU* (%)	Deviation vs Baseline	FPS (batch=1)
Baseline	67.3	0	60.1
Stripe-DW (m=7,14,21)	68.7	+1.4	58.9
Stripe-DW (no fusion)	68.2	+0.9	59.2
Single-scale (*m* = 21)	67.9	+0.6	59.5

We also performed an ablation study to compare an all-MLP decoder against a U-Net-style decoder while keeping backbone and all training hyperparameters fixed. For the U-Net-style decoder, we designed it as a three-stage combination of 3×3 convolutions with 2 times upsampling, similar to the standard U-Net model.

As indicated in Table 7, the all-MLP decoder requires fewer parameters and achieves a faster inference speed. Although the U-Net-style decoder delivers higher segmentation accuracy, the improvement in mIoU is marginal. These results demonstrate that the MLP-based decoder achieves a more favorable balance between accuracy and computational efficiency.

**Table 7:** Ablation results comparing all-MLP and U-Net-sytle decoder.

Decoder Type	Decoder Params (M)	mIoU (%)	FPS (batch=1)
All-MLP Decoder	4.2	68.7	58.9
U-Net-style Decoder	7.1	68.9	57.3

## Additional considerations

In this section, we provide a concise discussion of the practical application of the road surface sensing model in real-world scenarios, as well as challenges in developing a model that produces satisfactory results.

### Model’s performance in field experiment

**Fig 8:**
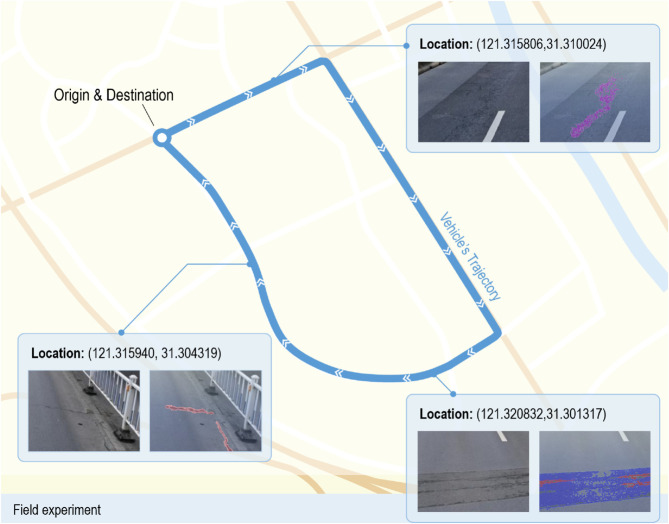
Field experiment of the proposed method.

Our proposed approach was implemented and evaluated in the context of an asphalt pavement environment in Shanghai, China. Fig 8 illustrates the field experiment scheme along with part of obtained results. In this experiment, a vehicle traversed a predefined route, while a computing terminal installed on-board captured real-time road surface images using a dashcam. Simultaneously, the terminal recorded the latitude and longitude coordinates of each image’s location with the assistance of GNSS equipment. The images were consistently fed into the model to do inference at a constant rate of 2 FPS.

Following meticulous frame-by-frame manual inspections, we observed that the proposed model demonstrates impressive proficiency in detecting subtle pavement diseases that often elude human observation. For the operating range, the model achieved highly accurate identification within a range of ap-proximately 30 meters, although instances of missed detection were noted beyond this distance.

### Application in the CAV scenario

In addition to supporting pavement maintenance, the system proposed in this paper also provides critical data for control of CAVs. Since an ego vehicle performing camera-based pavement distress detection typically has a limited detection range, directly using the pavement damage information it collects for driving control poses challenges. However, with the rapid advancements in V2V and V2X communication technologies, which enable seamless information sharing, the proposed method holds significant potential when applied to CAV and V2X environments.

**Fig 9:**
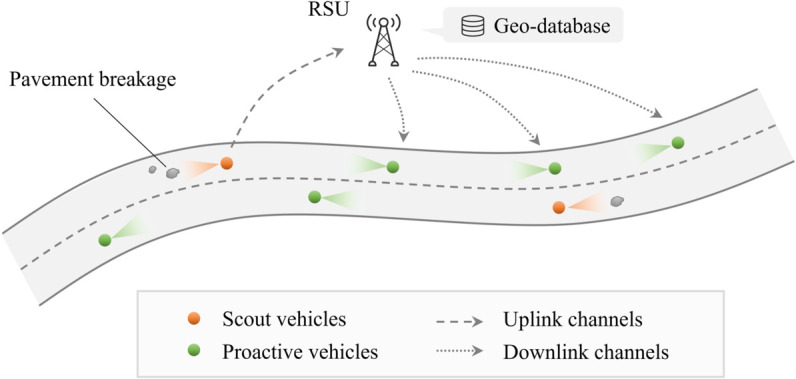
Pavement breakage detection in V2X environment.

As shown in Fig 9, within the V2X framework, the vehicle that first detects pavement damage is referred to as the “scout" vehicle, highlighted in orange. The scout vehicle transmits the spatial coordinates (via an onboard GPS module) and the details of the detected pavement distress to the roadside unit (RSU), which stores this data in a geospatial database. This database is accessible to all vehicles traveling on the same roadway. As other connected vehicles approach the location of the pavement damage, they can retrieve real-time information from the RSU, enabling them to take preventive actions, such as adjusting speed or changing lanes, to mitigate safety risks and improve driving comfort. These vehicles, depicted in blue in the figure, are designated as “proactive" vehicles. With the anticipated increase in CAV adoption, a growing number of vehicles will be able to serve as probe vehicles, ensuring the geospatial database is continuously updated with accurate pavement condition data.

### Challenges encountered in model training

During the training process of the pavement distress segmentation model, several challenges can arise, including class imbalance, image interference, and subpar segmentation outcomes. In practical scenarios, the occurrence frequency of different types of pavement distresses varies considerably, resulting in a scarcity of samples for certain distress categories within the collected dataset. For instance, the presence of potholes in the collected data is typically limited as they are often promptly treated, making it challenging to capture the feature of unpatched potholes accurately. Consequently, the identifi-cation of pavement distresses faces significant data imbalance. Addressing this issue, approaches such as focal loss [72], which considers sample weights, have been recommended, but their application in our experiments did not yield noticeable improvement. The utilization of generative models to amplify the sample size of rare disease types emerges as a promising approach that warrants empirical evaluation.

Moreover, vision-based sensing approaches can be perturbed by image in-terferences such as roadside trees and shadows from street lamps, which bear similarity to pavement distress types like cracks. To mitigate this situation, incorporating a shadow removal model (e.g., [73]) for image preprocessing presents a viable solution. Furthermore, the presence of edge jaggedness in the segmentation mask introduces errors in the pavement distress segmentation as observed in the results of numerical experiments. Although some studies [74] have proposed potential solutions, effectively tackling these challenges remains an ongoing endeavor for image segmentation models.

## Conclusion

Pavement condition sensing plays a crucial role in ensuring safe and comfortable driving within the automatic driving system. In this paper, we present a pavement condition semantic segmentation model based on a deep encoder-decoder neural network architecture with a spatial attention mechanism. The model is capable of identifying seven categories of pavement features, including cracks, potholes, and other surface irregularities, thereby enabling self-driving vehicles to perceive road surface conditions in real time and take appropriate actions to maintain smooth driving.

The encoder employs a multi-scale feature extraction network built on convolutional modules, enhanced with convolutional attention mechanisms to improve feature representation. The decoder adopts an MLP-based design, which reduces model complexity and accelerates inference. Numerical experiments demonstrate that the proposed model achieves strong performance, with an *mIoU* index of 68.7%. Notably, the *IoU* values for utility covers and expansion joints reach 87.3% and 71.6%, respectively, whereas cracks and alligator cracks yield comparatively lower values of 57.7% and 58.4%. Simultaneously, the proposed model achieves a balanced trade-off between accuracy and computational efficiency.

The proposed segmentation framework, which relies on onboard cameras, is distinguished by its cost-effectiveness and real-time processing capability. Nonetheless, in dense traffic scenarios, visibility of pavement conditions can be obstructed, potentially delaying timely damage detection. This limitation may be alleviated in CAV environments or through integration with complementary sensing technologies to enhance detection reliability. At the methodological level, the model’s accuracy requires further improvement, and the issue of performance discrepancies across different categories of pavement distresses remains to be addressed.

Despite outperforming baseline models, there remains scope for improvement in accuracy due to the similarity of different categories of distresses, dynamic blurring of images, change in illumination, shadows, and many other factors. Additionally, since the model was trained exclusively on asphalt pavement data, it is essential to evaluate its performance on pavement composed of other materials, e.g., concrete pavement. Conducting such comparative analyses will provide valuable insights into the model’s generalization capability across different pavement types. Furthermore, the process of annotating data for semantic segmentation entails significant costs. Therefore, the reduction of annotation requirements through the implementation of semi-supervised methods remains an ongoing issue in this field.

## References

[1] Amini B, Tehrani SS. Simultaneous effects of salted water and water flow on asphalt concrete pavement deterioration under freeze–thaw cycles. International Journal of Pavement Engineering. 2012;15(5):383–91. doi: 10.1080/10298436.2012.677844

[2] Anastasopoulos PC, Mannering FL, Shankar VN, Haddock JE. A study of factors affecting highway accident rates using the random-parameters tobit model. Accid Anal Prev. 2012;45:628–33. doi: 10.1016/j.aap.2011.09.015 22269550

[3] Ma N, Fan J, Wang W, Wu J, Jiang Y, Xie L, et al. Computer vision for road imaging and pothole detection: A state-of-the-art review of systems and algorithms. Transportation Safety and Environment. 2022;4(4). doi: 10.1093/tse/tdac026

[4] Kreidieh AR, Wu C, Bayen AM. Dissipating stop-and-go waves in closed and open networks via deep reinforcement learning. In: 2018 21st international conference on intelligent transportation systems (ITSC), 2018. 1475–80. 10.1109/itsc.2018.8569485

[5] Chen X, Ma H, Wan J, Li B, Xia T. Multi-view 3d object detection network for autonomous driving. In: Proceedings of the IEEE conference on computer vision and pattern recognition; 2017. p. 1907–15.

[6] Feng D, Harakeh A, Waslander SL, Dietmayer K. A review and comparative study on probabilistic object detection in autonomous driving. IEEE Trans Intell Transport Syst. 2022;23(8):9961–80. doi: 10.1109/tits.2021.3096854

[7] Arnold E, Al-Jarrah OY, Dianati M, Fallah S, Oxtoby D, Mouzakitis A. A survey on 3D object detection methods for autonomous driving applications. IEEE Trans Intell Transport Syst. 2019;20(10):3782–95. doi: 10.1109/tits.2019.2892405

[8] Sun L. Structural behavior of asphalt pavements: Intergrated analysis and design of conventional and heavy duty asphalt pavement. Butterworth-Heinemann; 2016.

[9] Mallick RB, El-Korchi T. Pavement engineering: Principles and practice. CRC Press; 2008.

[10] Vaswani A, Shazeer N, Parmar N, Uszkoreit J, Jones L, Gomez AN, et al. Attention is all you need. Adv Neural Inform Process Syst. 2017;30.

[11] Ronneberger O, Fischer P, Brox T. U-net: Convolutional networks for biomedical image segmentation. In: Medical image computing and computer-assisted intervention–MICCAI 2015 : 18th international conference, Munich, Germany, October 5–9, 2015, proceedings, part III 18. Springer; 2015. p. 234–41.

[12] Badrinarayanan V, Kendall A, Cipolla R. SegNet: A deep convolutional encoder-decoder architecture for image segmentation. IEEE Trans Pattern Anal Mach Intell. 2017;39(12):2481–95. doi: 10.1109/TPAMI.2016.2644615 28060704

[13] Guo MH, Lu CZ, Hou Q, Liu Z, Cheng MM, Hu SM. Segnext: Rethinking convolutional attention design for semantic segmentation. Adv Neural Inform Process Syst. 2022;35:1140–56.

[14] Woo S, Park J, Lee JY, Kweon IS. CBAM: Convolutional block attention module. In: Proceedings of the European conference on computer vision (ECCV); 2018. p. 3–19.

[15] Xie E, Wang W, Yu Z, Anandkumar A, Alvarez JM, Luo P. SegFormer: Simple and efficient design for semantic segmentation with transformers. Adv Neural Inform Process Syst. 2021;34:12077–90.

[16] El Hakea AH, Fakhr MW. Recent computer vision applications for pavement distress and condition assessment. Autom Constr. 2023;146:104664. doi: 10.1016/j.autcon.2022.104664

[17] Maeda H, Sekimoto Y, Seto T, Kashiyama T, Omata H. Road damage detection using deep neural networks with images captured through a smartphone. arXiv preprint arXiv:180109454. 2018.

[18] Eisenbach M, Stricker R, Seichter D, Amende K, Debes K, Sesselmann M, et al. How to get pavement distress detection ready for deep learning? A systematic approach. In: 2017 international joint conference on neural networks (IJCNN); 2017. p. 2039–47. 10.1109/ijcnn.2017.7966101

[19] Stricker R, Eisenbach M, Sesselmann M, Debes K, Gross H-M. Improving visual road condition assessment by extensive experiments on the extended GAPs dataset. In: 2019 International joint conference on neural networks (IJCNN); 2019. p. 1–8. 10.1109/ijcnn.2019.8852257

[20] Xu W, Tang Z, Zhou J, Ding J. Pavement crack detection based on saliency and statistical features. In: 2013 IEEE international conference on image processing; 2013. p. 4093–7. 10.1109/icip.2013.6738843

[21] Yousaf MH, Azhar K, Murtaza F, Hussain F. Visual analysis of asphalt pavement for detection and localization of potholes. Adv Eng Inform. 2018;38:527–37. doi: 10.1016/j.aei.2018.09.002

[22] Hoang N-D, Nguyen Q-L, Tien Bui D. Image processing–based classification of asphalt pavement cracks using support vector machine optimized by artificial bee colony. J Comput Civ Eng. 2018;32(5). doi: 10.1061/(asce)cp.1943-5487.0000781

[23] Shi Y, Cui L, Qi Z, Meng F, Chen Z. Automatic road crack detection using random structured forests. IEEE Trans Intell Transport Syst. 2016;17(12):3434–45. doi: 10.1109/tits.2016.2552248

[24] Chun P, Izumi S, Yamane T. Automatic detection method of cracks from concrete surface imagery using two-step light gradient boosting machine. Comput Aided Civil Eng. 2020;36(1):61–72. doi: 10.1111/mice.12564

[25] Ren S, He K, Girshick R, Sun J. Faster R-CNN: Towards real-time object detection with region proposal networks. Adv Neural Inform Process Syst. 2015;28. 10.1109/TPAMI.2016.2577031 27295650

[26] Redmon J, Farhadi A. YOLOv3: An incremental improvement. arXiv preprint. 2018. 10.48550/arXiv.1804.02767

[27] Du Y, Pan N, Xu Z, Deng F, Shen Y, Kang H. Pavement distress detection and classification based on YOLO network. Int J Pavement Eng. 2020;22(13):1659–72. doi: 10.1080/10298436.2020.1714047

[28] Zeng J, Zhong H. YOLOv8-PD: An improved road damage detection algorithm based on YOLOv8n model. Sci Rep. 2024;14(1):12052. doi: 10.1038/s41598-024-62933-z 38802524 PMC11130172

[29] Doshi K, Yilmaz Y. Road damage detection using deep ensemble learning. In: 2020 IEEE international conference on big data; 2020. p. 5540–4.

[30] Wang S, Chen X, Dong Q. Detection of asphalt pavement cracks based on vision transformer improved YOLO V5. J Transport Eng, Part B: Pavements. 2023;149(2):04023004.

[31] Huyan J, Li W, Tighe S, Zhai J, Xu Z, Chen Y. Detection of sealed and unsealed cracks with complex backgrounds using deep convolutional neural network. Autom Constr. 2019;107:102946. doi: 10.1016/j.autcon.2019.102946

[32] Yang F, Zhang L, Yu S, Prokhorov D, Mei X, Ling H. Feature pyramid and hierarchical boosting network for pavement crack detection. IEEE Trans Intell Transport Syst. 2020;21(4):1525–35. doi: 10.1109/tits.2019.2910595

[33] Naddaf-Sh S, Naddaf-Sh M-M, Kashani AR, Zargarzadeh H. An efficient and scalable deep learning approach for road damage detection. In: 2020 IEEE international conference on big data (big data); 2020. p. 5602–8. 10.1109/bigdata50022.2020.9377751

[34] Zhang Y, Liu C. Real-time pavement damage detection with damage shape adaptation. IEEE Trans Intell Transport Syst. 2024;25(11):18954–63. doi: 10.1109/tits.2024.3416508

[35] Lin C, Tian D, Duan X, Zhou J, Zhao D, Cao D. DA-RDD: Toward domain adaptive road damage detection across different countries. IEEE Trans Intell Transport Syst. 2023;24(3):3091–103. doi: 10.1109/tits.2022.3221067

[36] Yarram S, Varma G, Jawahar CV. City-scale road audit system using deep learning. In: 2018 IEEE/RSJ international conference on intelligent robots and systems (IROS); 2018. p. 635–40. 10.1109/iros.2018.8594363

[37] Liu W, Huang Y, Li Y, Chen Q. FPCNet: Fast pavement crack detection network based on encoder-decoder architecture. arXiv preprint arXiv:190702248. 2019.

[38] Zhang A, Wang KCP, Li B, Yang E, Dai X, Peng Y, et al. Automated pixel-level pavement crack detection on 3D asphalt surfaces using a deep-learning network. Comput Aided Civil Eng. 2017;32(10):805–19. doi: 10.1111/mice.12297

[39] Long J, Shelhamer E, Darrell T. Fully convolutional networks for semantic segmentation. In: 2015 IEEE conference on computer vision and pattern recognition (CVPR); 2015. p. 3431–40. 10.1109/cvpr.2015.7298965 27244717

[40] Fei Y, Wang KCP, Zhang A, Chen C, Li JQ, Liu Y, et al. Pixel-level cracking detection on 3D asphalt pavement images through deep-learning-based CrackNet-V. IEEE Trans Intell Transport Syst. 2020;21(1):273–84. doi: 10.1109/tits.2019.2891167

[41] Huyan J, Li W, Tighe S, Xu Z, Zhai J. CrackU-net: A novel deep convolutional neural network for pixelwise pavement crack detection. Struct Control Health Monit. 2020;27(8). doi: 10.1002/stc.2551

[42] Lau SLH, Chong EKP, Yang X, Wang X. Automated pavement crack segmentation using U-net-based convolutional neural network. IEEE Access. 2020;8:114892–9. doi: 10.1109/access.2020.3003638

[43] Jiang L, Xie Y, Ren T. A deep neural networks approach for pixel-level runway pavement crack segmentation using drone-captured images. arXiv preprint. 2020. https://doi.org/arXiv:200103257

[44] Majidifard H, Adu-Gyamfi Y, Buttlar WG. Deep machine learning approach to develop a new asphalt pavement condition index. Constr Build Mater. 2020;247:118513. doi: 10.1016/j.conbuildmat.2020.118513

[45] Ye G, Li S, Zhou M, Mao Y, Qu J, Shi T, et al. Pavement crack instance segmentation using YOLOv7-WMF with connected feature fusion. Autom Constr. 2024;160:105331. doi: 10.1016/j.autcon.2024.105331

[46] Mei Q, Gül M. A cost effective solution for pavement crack inspection using cameras and deep neural networks. Constr Build Mater. 2020;256:119397. doi: 10.1016/j.conbuildmat.2020.119397

[47] Zhang K, Zhang Y, Cheng H-D. CrackGAN: Pavement crack detection using partially accurate ground truths based on generative adversarial learning. IEEE Trans Intell Transport Syst. 2021;22(2):1306–19. doi: 10.1109/tits.2020.2990703

[48] Liu H, Yang J, Miao X, Mertz C, Kong H. CrackFormer network for pavement crack segmentation. IEEE Trans Intell Transport Syst. 2023;24(9):9240–52. doi: 10.1109/tits.2023.3266776

[49] Majidifard H, Jin P, Adu-Gyamfi Y, Buttlar WG. Pavement image datasets: A new benchmark dataset to classify and densify pavement distresses. Transport Res Record: J Transport Res Board. 2020;2674(2):328–39. doi: 10.1177/0361198120907283

[50] Amhaz R, Chambon S, Idier J, Baltazart V. Automatic crack detection on two-dimensional pavement images: An algorithm based on minimal path selection. IEEE Trans Intell Transport Syst. 2016;17(10):2718–29. doi: 10.1109/tits.2015.2477675

[51] Zhang L, Yang F, Daniel Zhang Y, Zhu YJ. Road crack detection using deep convolutional neural network. In: 2016 IEEE international conference on image processing (ICIP), 2016. 3708–12. 10.1109/icip.2016.7533052

[52] Zou Q, Cao Y, Li Q, Mao Q, Wang S. CrackTree: Automatic crack detection from pavement images. Pattern Recogn Lett. 2012;33(3):227–38. doi: 10.1016/j.patrec.2011.11.004

[53] Rateke T, Justen KA, Von Wangenheim A. Road surface classification with images captured from low-cost camera – Road traversing knowledge (RTK) dataset. RITA. 2019;26(3):50–64. doi: 10.22456/2175-2745.91522

[54] Mazzini D, Napoletano P, Piccoli F, Schettini R. A novel approach to data augmentation for pavement distress segmentation. Comput Ind. 2020;121:103225. doi: 10.1016/j.compind.2020.103225

[55] Deng F, Jin J, Chen X, An Z, Du Y. Enhancing automated health monitoring of road infrastructure through a hierarchical robust deep learning approach. Struct Health Monitor. 2024. doi: 10.1177/14759217241270919

[56] Howard AG, Zhu M, Chen B, Kalenichenko D, Wang W, Weyand T, et al. Mobilenets: Efficient convolutional neural networks for mobile vision applications. arXiv preprint arXiv:170404861. 2017.

[57] Szegedy C, Vanhoucke V, Ioffe S, Shlens J, Wojna Z. Rethinking the inception architecture for computer vision. In: Proceedings of the IEEE conference on computer vision and pattern recognition; 2016. p. 2818–26.

[58] Hendrycks D, Gimpel K. Gaussian error linear units (gelus). arXiv preprint arXiv:160608415. 2016.

[59] Huang Y, Qiu C, Yuan K. Surface defect saliency of magnetic tile. Vis Comput. 2018;36(1):85–96. doi: 10.1007/s00371-018-1588-5

[60] Zhou Z, Siddiquee MMR, Tajbakhsh N, Liang J. UNet++: A nested U-net architecture for medical image segmentation. arXiv preprint arXiv:180710165. 2018. 10.1007/978-3-030-00889-5_1 PMC7329239 32613207

[61] Chen LC, Zhu Y, Papandreou G, Schroff F, Adam H. In: Proceedings of the European conference on computer vision (ECCV); 2018. p. 801–18.

[62] Zhao H, Shi J, Qi X, Wang X, Jia J. Pyramid scene parsing network. In: Proceedings of the IEEE conference on computer vision and pattern recognition; 2017. p. 2881–2890.

[63] Li X, Zhong Z, Wu J, Yang Y, Lin Z, Liu H. Expectation-maximization attention networks for semantic segmentation. In: Proceedings of the IEEE/CVF international conference on computer vision; 2019. p. 9167–76.

[64] Cheng B, Schwing A, Kirillov A. Per-pixel classification is not all you need for semantic segmentation. Adv Neural Inform Process Syst. 2021;34:17864–75.

[65] Zheng S, Lu J, Zhao H, Zhu X, Luo Z, Wang Y. Rethinking semantic segmentation from a sequence-to-sequence perspective with transformers. In: Proceedings of the IEEE/CVF conference on computer vision and pattern recognition; 2021. p. 6881–90.

[66] Cheng B, Misra I, Schwing AG, Kirillov A, Girdhar R. Masked-attention mask transformer for universal image segmentation. In: Proceedings of the IEEE/CVF conference on computer vision and pattern recognition; 2022. p. 1290–1299.

[67] Xu J, Xiong Z, Bhattacharyya SP. PIDNet: A real-time semantic segmentation network inspired by PID controllers. In: 2023 IEEE/CVF conference on computer vision and pattern recognition (CVPR); 2023. p. 19529–39. 10.1109/cvpr52729.2023.01871

[68] Sandler M, Howard A, Zhu M, Zhmoginov A, Chen L-C. MobileNetV2: Inverted residuals and linear bottlenecks. In: 2018 IEEE/CVF conference on computer vision and pattern recognition; 2018. p. 4510–20. 10.1109/cvpr.2018.00474

[69] Tan M, Le Q. Efficientnet: Rethinking model scaling for convolutional neural networks. In: International conference on machine learning; 2019. p. 6105–14.

[70] Wei C, Wang W, Yang W, Liu J. Deep retinex decomposition for low-light enhancement. arXiv preprint. 2018. 10.48550/arXiv.1808.04560

[71] Jiang Y, Gong X, Liu D, Cheng Y, Fang C, Shen X, et al. EnlightenGAN: Deep light enhancement without paired supervision. IEEE Trans Image Process. 2021;30:2340–9. doi: 10.1109/TIP.2021.3051462 33481709

[72] Lin TY, Goyal P, Girshick R, He K, Dollár P. Focal loss for dense object detection. In: Proceedings of the IEEE international conference on computer vision; 2017. p. 2980–8.

[73] Jin Y, Sharma A, Tan RT. DC-shadownet: Single-image hard and soft shadow removal using unsupervised domain-classifier guided network. In: Proceedings of the IEEE/CVF international conference on computer vision; 2021. p. 5027–36.

[74] Kirillov A, Wu Y, He K, Girshick R. Pointrend: Image segmentation as rendering. In: Proceedings of the IEEE/CVF conference on computer vision and pattern recognition; 2020. p. 9799–808.

